# Draft Genome Sequence of a Meningitic Isolate of *Cronobacter sakazakii* Clonal Complex 4, Strain 8399

**DOI:** 10.1128/genomeA.00833-13

**Published:** 2013-10-10

**Authors:** Naqash Masood, Karen Moore, Audrey Farbos, Sumyya Hariri, Colin Block, Konrad Paszkiewicz, Ben Dickins, Alan McNally, Stephen Forsythe

**Affiliations:** Pathogen Research Group, School of Science and Technology, Nottingham Trent University, Nottingham, United Kingdoma; Wellcome Trust Biomedical Informatics Hub, Biosciences, University of Exeter, Exeter, United Kingdomb; Department of Clinical Microbiology and Infectious Diseases, Hadassah University Hospital, Jerusalem, Israelc

## Abstract

The *Cronobacter sakazakii* clonal lineage defined as clonal complex 4 (CC4), composed of nine sequence types, is associated with severe cases of neonatal meningitis. To date, only closely related *C. sakazakii* sequence type 4 (ST4) strains have been sequenced. *C. sakazakii* strain 8399, isolated from a case of neonatal meningitis, was sequenced as the first non-ST4 *C. sakazakii* strain.

## GENOME ANNOUNCEMENT

The *Cronobacter* genus is associated with severe human infections, such as meningitis, septicemia, and necrotizing enterocolitis (1). A multilocus sequence typing scheme has been established for the genus, and >200 sequence types (STs) and 37 clonal complexes have been defined (1). Previous studies of *Cronobacter* have revealed a strong association of the *C. sakazakii* clonal complex 4 (CC4) strains with neonatal meningitis (2, 3). Therefore, an improved understanding of *C. sakazakii* CC4 is warranted to understand its pathogenicity. This study sequenced *C. sakazakii* 8399, as it was isolated in 2000 from the cerebral spinal fluid of a premature baby with resulting severe anatomical damage to the brain and neurological deficit (4). This sequence can be compared with those of more recently isolated and sequenced *C. sakazakii* strains (5).

*C. sakazakii* 8399 DNA was extracted from 1-day cultures using the GenElute bacterial genome kit (Sigma-Aldrich, USA) and sequenced using an Illumina HiSeq 2500 system. A total of 6,139,848 high-quality paired-end reads of 150 bp in length, with 30-fold coverage were generated. *De novo* assembly was performed with Velvet (6). Further annotation used the SEED-based automated annotation system provided by the RAST server (7).

The genome of *C. sakazakii* 8399 is 4,662,173 bp in length, with a G+C content of 56.6%. The genome is distributed in 44 contigs with 4,359 coding sequences (CDSs) and 92 RNAs.

The CDSs include genes associated with iron acquisition, stress responses, heavy metal resistance (to arsenic, copper cobalt, zinc, and cadmium), and phages. Several virulence-associated traits, such as adhesins and sialic acid utilization, were also determined. These traits have previously been described in *Cronobacter* (5).

### Nucleotide sequence accession number.

The genome sequences of *C. sakazakii* 8399 have been deposited in GenBank under the accession no. AWSP00000000.
